# Increased Fecal Neopterin Parallels Gastrointestinal Symptoms in COVID-19

**DOI:** 10.14309/ctg.0000000000000293

**Published:** 2021-01-12

**Authors:** Felix Grabherr, Maria Effenberger, Alisa Pedrini, Lisa Mayr, Julian Schwärzler, Simon Reider, Barbara Enrich, Gernot Fritsche, Sophie Wildner, Rosa Bellmann-Weiler, Günter Weiss, Sabine Scholl-Bürgi, Thomas Müller, Alexander Moschen, Timon E. Adolph, Herbert Tilg

**Affiliations:** 1 Department of Internal Medicine I, Gastroenterology, Hepatology, Endocrinology & Metabolism, Medical University of Innsbruck, Innsbruck, Austria;; 2 Department of Internal Medicine II, Infectious Disease, Pulmonology & Rheumatology, Medical University of Innsbruck, Innsbruck, Austria;; 3 Department of Pediatrics I, Medical University of Innsbruck, Innsbruck, Austria.

## Abstract

**INTRODUCTION:**

Coronavirus disease (COVID-19) has spread from Wuhan, China, and become a worldwide pandemic. Most patients display respiratory symptoms but up to 50% report gastrointestinal symptoms. Neopterin is a surrogate marker for viral inflammation, and its production by macrophages is driven by interferon-γ.

**METHODS:**

We measured fecal neopterin in 37 hospitalized COVID-19 patients not requiring intensive care measures and 22 healthy controls.

**RESULTS:**

Fecal neopterin was elevated in stool samples from COVID-19 patients compared with that in samples from healthy controls. Especially, patients reporting gastrointestinal symptoms exhibited increased fecal neopterin values.

**DISCUSSION:**

COVID-19 is associated with an inflammatory immune response in the gastrointestinal tract.

## INTRODUCTION

Coronavirus disease (COVID-19), caused by the severe acute respiratory syndrome coronavirus 2 (SARS-CoV-2), has spread from Wuhan, Hubei Province, China, and become a worldwide pandemic. Although most patients display respiratory symptoms (1), initial studies reported that up to 50% of patients also have gastrointestinal (GI) symptoms such as diarrhea, vomiting, or nausea (2) such that Centers for Disease Control and Prevention integrated GI symptoms into the catalog of COVID-19 symptoms (3). More recent studies estimated that between 10% and 30% of patients experienced GI symptoms (4,5). SARS-CoV-2 RNA was detected in stool of patients (6) and SARS-CoV-2 infects and replicates in human intestinal epithelium (7), which is why a fecal–oral transmission could be possible. This is notable as 8 of 10 SARS-CoV-2 infected children exhibited polymerase chain reaction positive rectal swabs whereas nasopharyngeal tests were already negative (8). Recently, we could show that elevated fecal calprotectin, a reliable biomarker for activity and inflammation in inflammatory bowel disease (IBD) (9), correlates with serum interleukin-6 (a cytokine that indicates disease severity (10)) in hospitalized COVID-19 patients (6). These findings suggest an inflammatory response in the gut of COVID-19 patients. Neopterin is a pteridine derivate, which is produced mainly by macrophages on stimulation with interferon (IFN)-γ (11). Neopterin is particularly induced during viral infections, such as Epstein-Barr virus and cytomegalovirus. Fecal neopterin (fNEO) is also a biomarker that adequately reflects endoscopic activity in patients with IBD (12,13). In this study, we evaluated whether fNEO is elevated in COVID-19 patients compared with that of healthy controls, which would provide evidence that SARS-CoV-2 elicits immune activation and inflammation in the gut.

## METHODS

**Figure 1.**
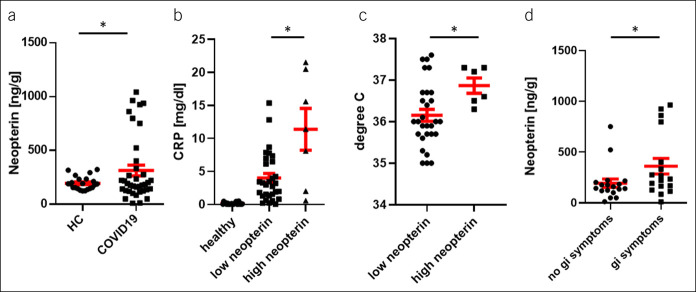
(**a**) The fNEO levels (ng/g) in healthy controls (HCs) (n = 22) and COVID-19 patients (n = 37), *P* = 0.038; note a subgroup of elevated fNEO samples above 614.7 ng/g. (**b**) C-reactive protein levels (mg/dL) in HCs and COVID-19 patients with high or low fNEO (cutoff 614.7 ng/g), *P* = 0.026. (**c**) Body temperature in degree celsius of the low and high fNEO group, *P* = 0.036. (**d**) The fNEO values in patients with and without GI symptoms; note increased values in the group presenting with GI symptoms, *P* = 0.049. Data shown as mean ± SEM; statistic tests used: 1-tailed *t* test for (**a**), unpaired *t* test for (**c**), Mann–Whitney test for (**b** and **d**). COVID-19, coronavirus disease 2019; fNEO, fecal neopterin; GI, gastrointestinal.

We collected fecal samples from 37 hospitalized COVID-19 patients at the University Hospital of Innsbruck, Austria, who did not require intensive care measures; 22 healthy subjects served as healthy controls. Diarrhea, defined as loose stools >3 times per day, nausea, and vomiting >1 time per day were included as GI symptoms in this study. We excluded other causes of acute GI infection by stool analysis for common viral, bacterial, parasitic, and protozoan pathogens in all patients with diarrhea, and no other chronic intestinal disease was documented for any patient. Infection with SARS-CoV-2 was confirmed by polymerase chain reaction of nasopharyngeal swab, recommended by the Centers for Disease Control and Prevention (Atlanta, GA). For collecting the swab systems, the Xpert Nasopharyngeal Sample Collection Kit (Cepheid, Sunnyvale, CA) was used. Informed consent was obtained from all patients, and ethics approval was granted as an amendment to AN2017-0016369/4.21. Supernatant for fNEO ELISA measurement of fecal samples was isolated using the extraction buffer from the Calprest Kit (Eurospital SpA, Triest, Italy). Fecal isolation was performed to the manufacturer’s protocol. In brief, fecal samples were diluted in extraction buffer and vortexed for 30 seconds; afterward, homogenization was performed for 30 minutes on a tube rotator. Homogenized samples were centrifuged at 10,000*g* for 20 minutes. Supernatant was used for quantification of neopterin with the Neopterin Elisa Kit (RE59321; Tecan, IBL International, Hamburg, Germany), according to the manufacturer's recommendation. Patients were classified as high or low fNEO group using the 80% of patients as cutoff, allowing a 100% specificity and 19% sensitivity. For statistical analysis, 1- or 2-tailed *t* test or Mann–Whitney test was used (as appropriate) to assess statistical significance. A 1-tailed *t* test was used in Figure 1a, as we predicted, partly based on the findings from the study by Husain et al. and our fecal calprotectin findings (6,13) that COVID-19 patients exhibit higher fNEO concentrations. A *P* value of <0.05 was assumed to be statistically significant. Statistical analysis was performed with GraphPad Prism V8 (San Diego, CA).

## RESULTS

Characteristics, frequency of GI symptoms, and biochemical parameters of 37 COVID-19 patients included in this study are presented in Table 1. In brief, 11 women and 26 men COVID-19 patients, with a median age of ∼62 years and evidence of systemic inflammation (indicated by C-reactive protein) of which 17 displayed GI symptoms (diarrhea and/or nausea and/or vomiting) were included in this study. In this cohort, fNEO values were elevated when compared with those of healthy controls (Figure 1a, mean 314.6 ng/g, SEM 50.0 vs mean 195.9 ng/g, SEM 12.9; *P* = 0.038; 95% confidence interval [CI] 213.1–416.0 vs 169.0–222.8). A disease-discriminating cutoff of 614.7 ng/g fNEO (i.e., the 80%, allowing a 100% specificity and 19% sensitivity) suggested that only a group of COVID-19 patients displayed elevated (>614.7 ng/g) fNEO concentrations in the stool. In this patient group, we observed significantly higher serum C-reactive protein concentration when compared with the low neopterin group (Figure 1b, mean 4.017 mg/dL, SEM 0.687 vs mean 11.39 mg/dL, SEM 3.160, *P* = 0.026; 95% CI 2.6–5.4 vs 3.7–19.1) and an elevated body temperature on the day of fecal sample collection (Figure 1c, mean 36.87°C, SEM 0.183 vs mean 36.15°C, SEM 0.142, *P* = 0.03; 95% CI 36.39–37.34 vs 35.86–36.44), indicating systemic inflammation. Notably, elevated fNEO concentration was particularly observed in patients with GI symptoms (Figure 1d, mean 194.6 ng/g, SEM 41.4 vs mean 362.1 ng/g, SEM 76.6; *P* = 0.049; 95% CI 107.3–281.8 vs 199.9–524.4). As such, COVID-19 patients displayed increased fNEO concentration, particularly during presentation of GI symptoms.

**Table 1.**
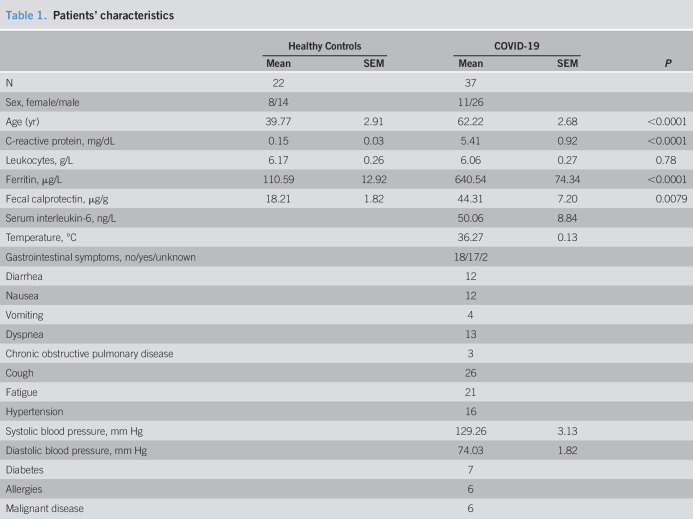
Patients' characteristics

	Healthy Controls		COVID-19		*P*
	Mean	SEM	Mean	SEM	
N	22		37		
Sex, female/male	8/14		11/26		
Age (yr)	39.77	2.91	62.22	2.68	<0.0001
C-reactive protein, mg/dL	0.15	0.03	5.41	0.92	<0.0001
Leukocytes, g/L	6.17	0.26	6.06	0.27	0.78
Ferritin, μg/L	110.59	12.92	640.54	74.34	<0.0001
Fecal calprotectin, μg/g	18.21	1.82	44.31	7.20	0.0079
Serum interleukin-6, ng/L			50.06	8.84	
Temperature, °C			36.27	0.13	
Gastrointestinal symptoms, no/yes/unknown			18/17/2		
Diarrhea			12		
Nausea			12		
Vomiting			4		
Dyspnea			13		
Chronic obstructive pulmonary disease			3		
Cough			26		
Fatigue			21		
Hypertension			16		
Systolic blood pressure, mm Hg			129.26	3.13	
Diastolic blood pressure, mm Hg			74.03	1.82	
Diabetes			7		
Allergies			6		
Malignant disease			6		

## DISCUSSION

GI symptoms occur in COVID-19 patients frequently (2,14,15), whereas mucosal disturbances in the gut are poorly understood. A recent study indicated that SARS-CoV-2 can infect and replicate in human enterocytes (7). Furthermore, it was shown that ACE2 and transmembrane serine protease 2, the 2 key proteins for cell entry of the SARS-CoV-2, are coexpressed on intestinal epithelial cells (16). Both findings indicate a potential route of infection of SARS-CoV-2 in the intestinal tract. We show that fNEO, a surrogate of a cellular viral immune response (17), is increased in fecal samples of COVID-19 patients, particularly in those with GI symptoms. Our findings underline the involvement of the GI tract in COVID-19 and go along with several recent studies showing frequent GI inflammation in COVID-19 (2,14,15). COVID-19 patients seem to display comparable (or slightly higher) fNEO values when compared with active IBD patients (12,13), suggesting that the COVID-19 inflammatory response in the gut is similarly pronounced compared to an IBD disease flare. Of interest, we did not see any difference in fNEO depending on which GI symptom patients where reporting.

Neopterin synthesis takes place almost exclusively in activated macrophages primarily under the control of IFNγ (11). COVID-19 is characterized by activation of innate immunity (10,18), which also involves the intestine indicated by the data of this study. Disease severity in COVID-19 correlates with the detection of various cytokines and chemokines including IFNγ (18). The release of a plethora of cytokines and chemokines by infected cells (such as enterocytes) might result in intestinal inflammation, which might underlie GI symptoms. Although this study has some limitations (small sample size, younger age of controls, and SARS-COV-2 RNA was only measurable in 7 of 20 samples), we believe that our findings together with previous reports collectively indicate that COVID-19 is a systemic viral disease, causing GI inflammation and an innate intestinal immune response.

## CONFLICTS OF INTEREST

**Guarantor of the article:** Herbert Tilg, MD.

**Specific author contributions:** Felix Grabherr, MD, and Maria Effenberger, MD, contributed equally to this article. F.G. and M.E.: prepared the manuscript and collected and analyzed data. T.E.A. and H.T.: coordinated the project and helped preparing the manuscript. A.P., L.M., J.S., S.R., and B.E.: processed and measured the fecal samples. S.S.B., T.M., and A.M.: provided access to clinical samples. G.F., S.W., R.B.-W., and G.W.: involved in patient care and provided access to clinical samples.

**Financial support:** This study is supported by the excellence initiative VASCage (Centre for Promoting Vascular Health in the Ageing Community), an R&D K-Centre (COMET programme—Competence Centers for Excellent Technologies) funded by the Austrian Ministry for Transport, Innovation and Technology, the Austrian Ministry for Digital and Economic Affairs, and the federal states Tyrol, Salzburg and Vienna (to H.T.). T.E. Adolph was supported by the Austrian Science Fund (FWF): FP33070-B.

**Potential competing interests:** None to report.

**Study Highlights**

**WHAT IS KNOWN**

✓ COVID-19 is a systemic disease involving the GI tract.
✓ Neopterin is a surrogate marker for viral inflammation.

**WHAT IS NEW HERE**

✓ Fecal neopterin is elevated in COVID-19 patients compared with that in healthy controls.
✓ Patients reporting gastrointestinal symptoms have higher fecal neopterin values.

**TRANSLATIONAL IMPACT**

✓ Fecal neopterin indicates intestinal inflammation in COVID-19.
